# Role of the Modified Glasgow Prognostic Score (mGPS) as a Prognostic Factor in Metastatic Colorectal Cancer

**DOI:** 10.7759/cureus.64916

**Published:** 2024-07-19

**Authors:** Horia Cotan, Cristian Iaciu, Cornelia Nitipir

**Affiliations:** 1 Oncology, Elias Emergency University Hospital, Bucharest, ROU; 2 Clinic of Oncology, Carol Davila University of Medicine and Pharmacy, Bucharest, ROU

**Keywords:** body mass index (bmi), inflammation, glasgow prognostic score, prognostic biomarkers, colorectal cancer

## Abstract

Background

This study aims to evaluate the prognostic significance of the modified Glasgow Prognostic Score (mGPS) in patients with metastatic colorectal cancer (mCRC).

Methodology

A retrospective analysis was conducted among 65 patients diagnosed with stage IV colorectal cancer who received treatment and follow-up at the Oncology Department of Elias Emergency University Hospital in Bucharest, Romania, from January 2016 to January 2024. Patient data were collected, including demographic information, tumor characteristics, and laboratory parameters. The mGPS was calculated based on serum albumin and C-reactive protein (CRP) levels. Patients were stratified into the following three mGPS categories: 0 (normal CRP and albumin), 1 (elevated CRP or hypoalbuminemia), and 2 (elevated CRP and hypoalbuminemia).

Results

Of the 65 patients included, 33 (50.8%) were male and 32 (49.2%) were female, with a mean age of 63.7 years. According to mGPS, 25 (38.5%) patients scored 0, 30 (46.2%) scored 1, and 10 (15.4%) scored 2. The median overall survival (OS) was 53 months (95% confidence interval (CI) = 23.512-82.488), and the median progression-free survival (PFS) was 23 months (95% CI = 19.244-26.756). Although numerical differences in the median PFS and OS were observed between treatment groups, these differences were not statistically significant (PFS: p = 0.292; OS: p = 0.5).

Conclusions

The mGPS is a useful prognostic tool in mCRC, providing insights into patient survival outcomes. However, further studies with larger sample sizes are needed to validate these findings and clarify the role of mGPS in guiding clinical decision-making for mCRC patients.

## Introduction

Colorectal cancer (CRC) is a major public health issue, ranking as the third most prevalent cancer worldwide with almost 2 million cases diagnosed in 2020, following breast and lung cancer [1]. In the same year, CRC accounted for 9.4% of all cancer-related deaths, totaling 935,173 deaths. These figures are projected to rise significantly, with estimates predicting 3.2 million new cases and 1.6 million deaths annually by 2040 [2]. Despite advancements in surgical techniques and adjuvant and neoadjuvant therapies, the five-year survival rate for CRC patients varies widely from 90% to 10%, depending on tumor characteristics, while the five-year median survival rate for metastatic CRC (mCRC) is around 30% [3].

One of the most compelling areas of clinical research is the host’s inflammatory response to tumors, which has been demonstrated to significantly influence cancer progression and metastasis. Inflammatory mediators, such as cytokines, increase vascular permeability, facilitating cancer cell entry into lymphatic and blood vessels. These mediators also promote tumor angiogenesis and attract immune cells that aid in tumor growth [4,5].

Among the cytokines involved in the systemic inflammatory response, interleukin-6 (IL-6) and transforming growth factor-β (TGF-β) are particularly significant. IL-6 enhances the production of acute-phase proteins such as C-reactive protein (CRP), reduces albumin production in the liver, promotes megakaryocyte development into platelets, and aids in neutrophil recruitment, ultimately increasing platelet numbers [6]. TGF-β, on the other hand, polarizes antitumoral neutrophils into protumoral ones, fostering a tumor-friendly immune microenvironment [7]. Numerous studies have highlighted the prognostic value of inflammatory biomarkers, leading to the development of scores such as the Glasgow Prognostic Score (GPS) [8], modified Glasgow Prognostic Score (mGPS) [9], neutrophil-to-lymphocyte ratio [10], platelet-to-lymphocyte ratio [11], and lymphocyte-to-monocyte ratio [12] for predicting survival in CRC patients.

The GPS was first introduced by Forrest et al. [13] in the context of non-small-cell lung cancer, where a retrospective study validated its prognostic value. Since then, numerous studies have confirmed GPS as a significant prognostic determinant in other cancers, including pancreatic, hepatocellular, and esophageal cancers [14]. Subsequently, researchers developed the mGPS, which also utilizes CRP and albumin levels as markers.

Despite its proven utility in various cancers, the effectiveness of mGPS in predicting CRC outcomes remains inconclusive. While mGPS has shown promise as a prognostic marker in several cancers, its role in CRC has been inconsistent, with some studies failing to establish a clear link between mGPS and CRC prognosis. This discrepancy highlights the need for further investigation to clarify the potential of mGPS in CRC prognostication and to determine whether it can serve as a reliable predictive tool for CRC outcomes.

The primary objective of this study is to explore and establish the prognostic and potential predictive role of mGPS, specifically in the context of mCRC. By focusing on mCRC, we aim to determine whether mGPS can provide valuable insights into patient outcomes and serve as a useful marker for guiding treatment decisions in metastatic cases.

## Materials and methods

Study population

All patients in this retrospective study received treatment and follow-up at the Oncology Department of Elias Emergency University Hospital in Bucharest, Romania, from January 2016 to January 2024. Each participant was diagnosed with stage IV CRC. The primary treatment for these patients involved palliative chemotherapy, with options including doublet chemotherapy (FOLFOX, FOLFIRI, or CAPEOX) combined with bevacizumab or anti-epidermal growth factor receptor (EGFR) antibodies such as cetuximab or panitumumab. Treatment choices were based on the primary tumor location and *RAS*/*BRAF* status. Notably, none of the stage IV patients in this study received immunotherapy.

The inclusion criteria required a confirmed CRC diagnosis through histopathological and immunohistochemical assessments and precise clinical staging. Pathological staging was conducted by an experienced pathologist using the American Joint Committee on Cancer TNM Staging Classification for Colon Cancer, 8th edition, 2017. Clinical staging involved comprehensive CT or MRI scans of the chest, abdomen, pelvis, and brain for symptomatic patients. Lesions suspected to be metastases but not definitively identified on imaging were biopsied for histopathological and immunohistochemical confirmation. Exclusion criteria included signs or symptoms of infection (e.g., elevated procalcitonin levels, leukocytosis, fever, malaise, abnormal chest radiography, or positive cultures from blood, urine, or pharyngeal exudate), immunocompromised status, autoimmune pathologies, ongoing corticosteroid therapy, and diagnoses of other synchronous cancers. Another exclusion criterion was applied wherein no patients operated on with curative intent were included in the analysis. Instead, the study concentrated exclusively on individuals exhibiting unresectable CRC.

**Figure 1:**
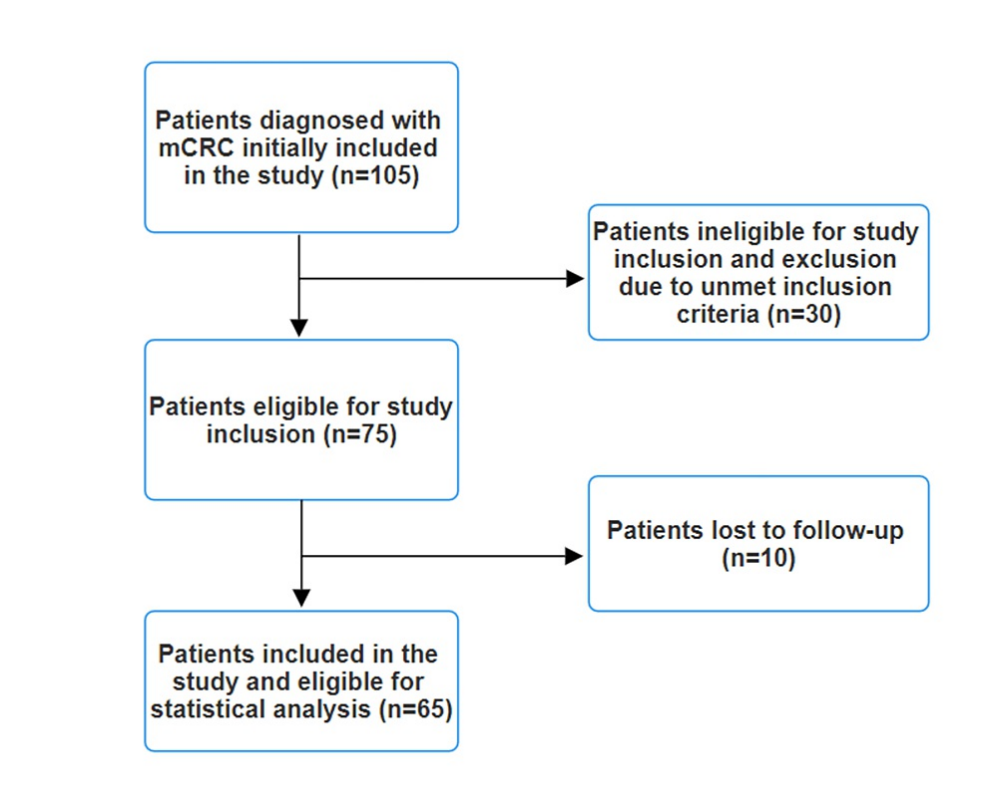
Study flowchart. mCRC = metastatic colorectal cancer

A total of 105 patients were screened for this study, while only 65 were eligible for statistical analysis after inclusion and exclusion criteria were applied (Figure 1).

Data collection

We collected data retrospectively, focusing on demographic information such as age, gender, and family history. Additionally, we analyzed tumor characteristics, including location, differentiation, TNM stage, molecular biomarkers, and laboratory parameters, such as complete blood count, serum albumin, CRP, carcinoembryonic antigen (CEA), and carbohydrate antigen 19-9 (CA 19-9).

The mGPS was determined using the methodology outlined in Table 1, which relies on two biomarkers, namely, serum albumin levels and CRP levels. Patients with both elevated CRP (>10 mg/L) and hypoalbuminemia (<35 g/L) were assigned a score of 2. Patients with only elevated CRP received a score of 1, while those with neither abnormality were assigned a score of 0.

**Table 1:** The modified Glasgow Prognostic Score (mGPS).

C-reactive protein levels	Serum albumin levels	Points allocated
≤10 mg/L	≥35 g/L	0
>10 mg/L	≥35 g/L	1
>10 mg/L	<35 g/L	2

We also recorded patients’ body mass index (BMI). We then classified patients into the following groups based on BMI: underweight (<18.5 kg/m^2^), normal weight (18.5-24.9 kg/m^2^), and overweight (>25 kg/m^2^).

Statistical analysis

Statistical analysis was conducted using SPSS software version 26.0 (IBM Corp., Armonk, NY, USA). Descriptive statistics reported patient and disease characteristics. Group differences were assessed using Pearson’s chi-squared or Fisher’s exact test for categorical variables, and the t-test or Wilcoxon rank-sum test for continuous variables. Median survival was evaluated using the Kaplan-Meier method and compared using the log-rank test. Univariate and multivariate Cox regression models analyzed the relationship between elevated mGPS levels and the risk of death or recurrence in CRC patients, adjusting for potential confounders.

Overall survival (OS) was calculated from diagnosis to death and progression-free survival (PFS) from treatment initiation to first progression per the RECIST criteria. The overall response rate (ORR) included partial or complete responses to the first line of therapy, with partial response, complete response, and stable disease considered favorable, and progressive disease unfavorable. Continuous variables were presented as mean and standard deviation (SD) or median and quartiles, while categorical variables were reported as counts (n) and percentages (%). A p-value less than 0.05 was considered statistically significant.

## Results

Patient characteristics

Out of 110 cases of mCRC patients treated with systemic therapy, only 65 were included in this analysis after the application of inclusion/exclusion criteria. There were 33 (50.8%) male patients and 32 (49.2%) female patients. The average age was 63.7 years, with the range of 37 to 81 years old. Most patients (n = 49; 73.4%) were microsatellite stable/proficient mismatch repair while only 16 (26.6%) patients were microsatellite instability-high/deficient mismatch repair. Treatment received varied depending on *RAS*/*BRAF* mutational status and location of the primary tumor. Most patients (n = 48; 73.8%) received doublet chemotherapy and an anti-EGFR agent, while 17 (26.2%) received doublet chemotherapy (FOLFOX/CAPEOX/FOLFIRI) and bevacizumab.

According to the mGPS, 25 (38.5%) patients had a score of 0 points, 30 (46.2%) patients reached a score of 1, and 10 (15.4%) patients reached a score of 2.

The median OS and PFS were 53 months (95% confidence interval (CI) = 23.512-82.488) and 23 months (95% CI = 19.244-26.756), respectively. Although there was a numerical difference in median PFS between treatment groups, it was not statistically significant (p = 0.292). Patients receiving doublet chemotherapy and bevacizumab had a median PFS of 36 months (95% CI = 11.262-58.074) compared to 23.5 months (95% CI = 19.244-26.756) for those receiving doublet chemotherapy and an anti-EGFR antibody. Similarly, OS showed a numerical difference but was statistically insignificant (p = 0.5). The longest OS was observed in patients receiving doublet chemotherapy and bevacizumab, with a median of 63 months (95% CI = 14.211-93.755) compared to 53 months (95% CI = 12.320-87.680) for those treated with doublet chemotherapy and an anti-EGFR antibody. Details are provided in Table 2.

**Table 2:** Baseline clinical characteristics of patients. CI = confidence interval; PFS = progression-free survival; OS = overall survival; mGPS = modified Glasgow Prognostic Score; ECOG = Eastern Cooperative Oncology Group; MSI-H/dMMR = microsatellite instability-high/deficient mismatch repair; MSS/pMMR = microsatellite stable/proficient mismatch repair; CEA = carcinoembryonic antigen; CA 19-9 = cancer antigen 19-9; BMI = body mass index; CHT = chemotherapy; EGFR = epidermal growth factor receptor

	Doublet CHT + anti-EGFR agent (n = 48)	Doublet CHT + bevacizumab (n = 17)
Age, years, mean (SD)	62.3 (±6.33)	64.6 (±3.41)
Gender, n (%)		
Male	24 (50%)	9 (45.9%)
Female	24 (50%)	8 (54.1%)
ECOG, n (%)		
0	31(64.5%)	10 (58.8%)
1	10 (20.8%)	4 (23.5%)
≥2	7 (14.6%)	3 (17.6%)
Primary tumor location, n (%)		
Right side	0 (0%)	12 (70.6%)
Left side	48 (100%)	5 (29.6%)
RAS mutational status, n (%)		
Wild-type	48 (100%)	9 (48.6%)
Mutant	0 (0%)	8 (51.4%)
BRAF mutational status, n (%)		
Wild-type	48 (100%)	14 (82.3%)
Mutant	0 (0%)	3 (17.7%)
MSI/MMR status, n (%)		
MSI-H/dMMR	8 (16.6%)	8 (47%)
MSS/pMMR	40 (83.4%)	9 (53%)
Tumor burden		
Extrahepatic extension	26 (54.2%)	6 (35.3%)
Confined to the liver	22 (45.8%)	11 (64.7%)
CEA		
Over the upper limit	22 (45.8%)	4 (23.5%)
Under the upper limit	26 (54.2%)	13 (76.5%)
CA 19-9		
Over the upper limit	4 (8.3%)	1 (5.9%)
Under the upper limit	44 (91.7%)	16 (94.1%)
mGPS		
Score 0	18 (37.5%)	7 (41.2%)
Score 1	21 (43.7%)	9 (53%)
Score 2	9 (18.8%)	1 (5.9%)
BMI		
BMI <18.5	14 (29.2%)	0 (0%)
BMI 18.5–24.9	25 (52%)	14 (82.3%)
BMI >25	9 (18.8%)	3 (17.7%)
Median PFS (months)	23.5 months (95% CI = 19.244-26.756)	36 months (95% CI = 11.262-58.074)
Median OS (months)	53 months (95% CI = 12.320-87.680)	63 months (95% CI = 14.211-93.755)

Clinical outcomes according to mGPS

**Figure 2:**
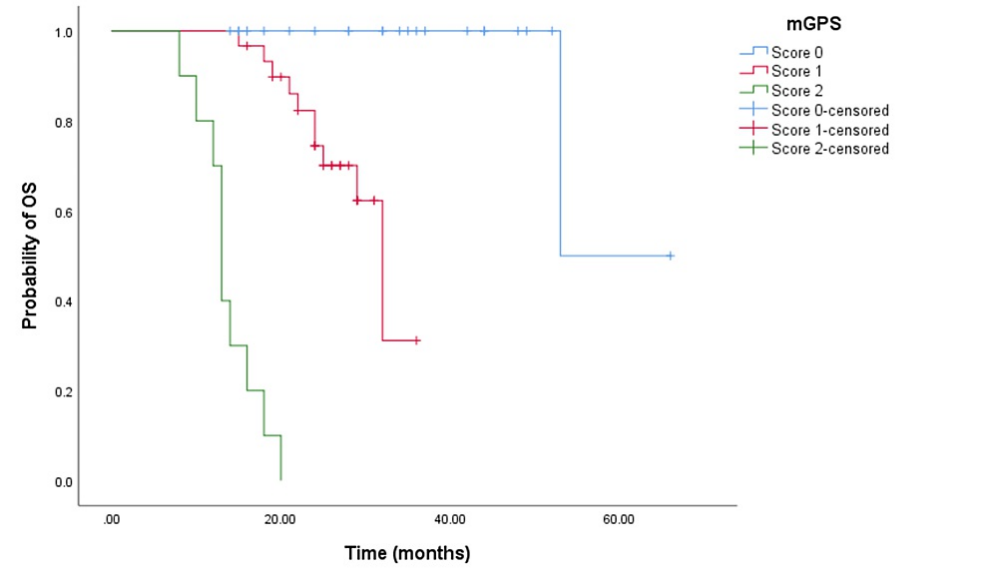
Comparative analysis of Kaplan-Meier curves between mGPS of 0, 1, and 2 patients for OS. OS = overall survival; mGPS = modified Glasgow Prognostic Score

**Figure 3:**
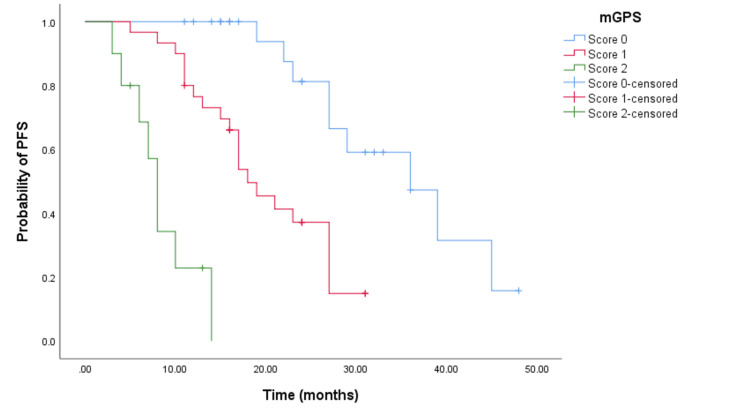
Comparative analysis of Kaplan-Meier curves between mGPS of 0, 1, and 2 patients for PFS. PFS = progression free survival; mGPS = modified Glasgow Prognostic Score

The Kaplan-Meier survival analysis demonstrated that CRC patients with a higher mGPS had significantly worse OS. Patients with an mGPS of 2 had a median OS of 13 months (95% CI = 11.988-14.012, p < 0.001) (see Figure 2) compared to 32 months (95% CI = 27.718-36.282, p < 0.001) for those with an mGPS of 1, and 53 months (95% CI not reached) for those with an mGPS of 0. Similarly, patients with a higher mGPS experienced shorter PFS. The median PFS for patients with an mGPS of 2 was eight months (95% CI = 6.635-9.365, p < 0.0001) (see Figure 3), whereas it was 18 months (95% CI = 14.163-21.837, p < 0.0001) (see Figure 3) for those with an mGPS of 1, and 36 months (95% CI = 25.453-46.546) for those with an mGPS of 0.

Clinical outcomes stratified by treatment according to mGPS

**Figure 4:**
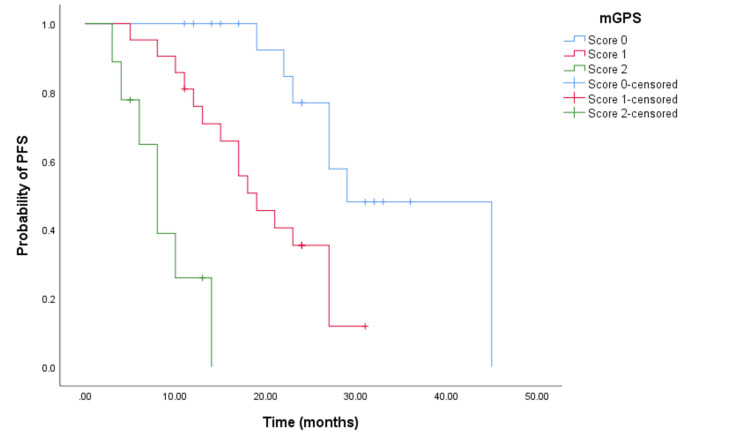
Comparative analysis of Kaplan-Meier curves between mGPS of 0, 1, and 2 patients for PFS in the cohort treated with doublet CHT and bevacizumab. PFS = progression free survival; mGPS = modified Glasgow Prognostic Score; CHT = chemotherapy

**Figure 5:**
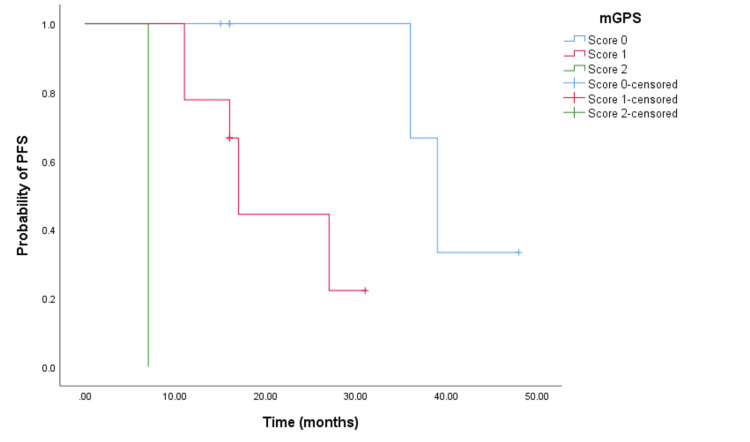
Comparative analysis of Kaplan-Meier curves between mGPS of 0, 1, and 2 patients for PFS in the cohort treated with doublet CHT and anti-EGFR agents. PFS = progression free survival; mGPS = modified Glasgow Prognostic Score; CHT = chemotherapy; EGFR = epidermal growth factor receptor

In the cohort receiving doublet chemotherapy and bevacizumab, significant differences in PFS times were observed among patients with mGPS scores of 0, 1, and 2. Specifically, the PFS times were 39 months (95% CI = 34.199-27.527, p < 0.001) (see Figure 4), 17 months (95% CI = 15.152-18.848, p < 0.001) (see Figure 4), and seven months (95% CI not reached) respectively. Similarly, for the cohort treated with doublet chemotherapy and an anti-EGFR agent, the PFS times significantly varied with mGPS scores of 0, 1, and 2, showing 29 months (95% CI = 19.793-38.207, p < 0.001) (see Figure 5), 19 months (95% CI = 13.248-24.752, p < 0.001 (see Figure 5), and eight months (95% CI = 5.381-10.619, p < 0.001) (see Figure 5), respectively.

**Figure 6:**
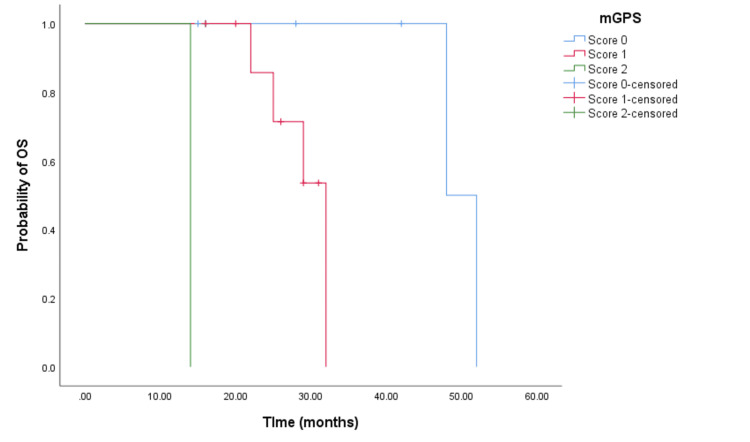
Comparative analysis of Kaplan-Meier curves between mGPS of 0, 1, and 2 patients for OS in the cohort treated with doublet CHT and anti-EGFR agents. OS = overall survival; mGPS = modified Glasgow Prognostic Score; CHT = chemotherapy; EGFR = epidermal growth factor receptor

**Figure 7:**
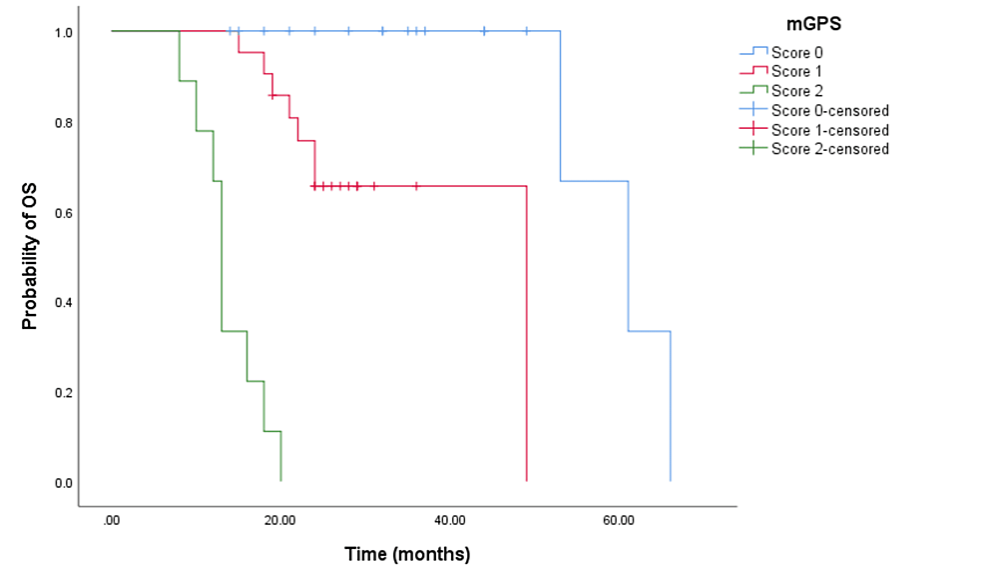
Comparative analysis of Kaplan-Meier curves between mGPS of 0, 1, and 2 patients for OS in the cohort treated with doublet CHT and bevacizumab. OS = overall survival; mGPS = modified Glasgow Prognostic Score; CHT = chemotherapy

In the cohort receiving doublet chemotherapy and bevacizumab, OS times also showed significant differences based on mGPS scores of 0, 1, and 2. The OS times were 48 months (95% CI = 41.229-51.822, p < 0.001) (see Figure 6), 32 months (95% CI = 25.789-34.283, p < 0.001) (see Figure 6), and 14 months (95% CI not reached), respectively. In the cohort treated with doublet chemotherapy and an anti-EGFR agent, OS times varied significantly with mGPS scores of 0, 1, and 2, displaying 61 months (95% CI = 48.197-73.803, p < 0.001) (see Figure 7), 49 months (95% CI = 32.832-55.532, p < 0.001) (see Figure 7), and 13 months (95% CI = 12.076-13.924, p < 0.001) (see Figure 7), respectively.

Multivariate analysis of the entire cohort

The prognostic value of mGPS has been confirmed in both the doublet chemotherapy and anti-EGFR agent cohort as well as in the doublet chemotherapy and bevacizumab cohort through multivariate analysis (Cox regression). The hazard ratio (HR) for death among patients with a mGPS of 2 was 8.127 (95% CI = 3.241-12.239, p < 0.001) (Table 3), while the HR for cancer progression was 5.933 (95% CI = 4.762-10.338, p < 0.001) (Table 3).

**Table 3:** Multivariate Cox regression analyses to identify predictors for increased risk of death and tumor progression considering mGPS as an independent variable. Statistically significant at p-values <0.05. CI = confidence interval; PFS = progression-free survival; HR = hazard ratio; OS = overall survival; mGPS = modified Glasgow Prognostic Score; ECOG = Eastern Cooperative Oncology Group; MSI-H/dMMR = microsatellite instability-high/deficient mismatch repair; MSS/pMMR = microsatellite stable/proficient mismatch repair; ref. = reference

Test variables	OS HR (95% CI)	P-value	PFS HR (95% CI)	P-value
mGPS 0 (ref.)/mGPS 1	4.091(2.241-7.469)	<0.001	4.799 (2.762-8.338)	<0.001
mGPS 0 (ref.)/mGPS 2	8.127 (3.241-12.239)	<0.001	5.933 (4.762-10.338)	<0.001
BRAF mutational status wild-type (ref.)/mutant	1.520 (1.340-1.781)	0.002	1.173 (1.046-1.259)	0.383
RAS mutational status wild-type (ref.)/mutant	1.602 (1.357-1.934)	0.004	1.371 (1.180-1.582)	0.459
ECOG 0-1 (ref.)/2	1.490 (1.089-2.038)	<0.001	1.428 (1.207-1.639)	0.087
Microsatellite instability MSI-H/dMMR (ref.)/MSS/pMMR	1.310 (0.672-4.222)	0.128	1.503 (1.249-1.764)	0.006
Tumor burden confined to liver (ref.)/extrahepatic extension	2.406 (1.905-2.951)	0.257	1.621 (1.308-1.937)	0.216
Carcinoembryonic antigen low (ref.)/high	1.215 (1.109-1.403)	<0.001	1.754 (1.403-2.096)	0.003
Cancer antigen 19-9 low (ref.)/high	1.057 (0.455-2.729)	0.433	1.269 (1.104-2.203)	0.247
BMI 18.5–24.9 (ref.)/BMI <18.5	1.878 (1.344-2.755)	0.001	1.739 (1.288-2.125)	0.005
BMI 18.5–24.9 (ref.)/BMI >25	0.977 (0.844-1.225)	0.391	1.003 (0.977-1.274)	0.493
Age <65 years (ref.)/>65 years	1.313 (1.145-1.535)	0.377	1.392 (1.162-1.489)	0.698
Gender female (ref.)/male	1.447 (0.849-2.466)	0.174	1.643 (1.319-1.982)	0.195
Location of primary tumor left side (ref.)/right side	1.110 (0.772-1.943)	0.324	1.518 (1.268-1.736)	0.229

We also examined the ORR categorized by mGPS. We defined a favorable outcome as a partial response, a complete response, or a stable disease, and an unfavorable outcome as a progressive disease. The majority of patients with mGPS of 2 (n = 8, 80%) (see Table 4) showed an unfavorable response, whereas the majority of patients with mGPS of 1 (n = 25, 83.3%) (see Table 4) exhibited a favorable response and all (n = 25, 100%) patients with mGPS of 0 exhibited a favorable response.

**Table 4:** Association between mGPS and ORR. ORR = overall response rate; mGPS = modified Glasgow Prognostic Score; CR = complete response; PR = partial response; SD = stable disease; PD = progressive disease

ORR	mGPS 0	mGPS 1	mGPS 2
Favorable response (CR + PR + SD) (n, %)	25 (100%)	25 (83.3%)	2 (20%)
Unfavorable response (PD) (n, %)	0 (0%)	5 (16.7%)	8 (80%)

We evaluated the correlation between BMI and mGPS. There was a significant correlation between BMI and mGPS, especially among mGPS 2 patients, with a majority (n = 6, 60%) of mGPS 2 patients being underweight. Data are shown in Table 5.

**Table 5:** Correlation between mGPS and BMI. Statistically significant at p-values <0.05. BMI = body mass index; mGPS = modified Glasgow Prognostic Score

BMI (kg/m^2^)	mGPS 0	mGPS 1	mGPS 2	P-value
<18.5 (n, %)	4 (16%)	4 (13.3%)	6 (60%)	0.001
18.5–24.9 (n, %)	15 (60%)	20 (66.7%)	4 (40%)	
>25 (n, %)	6 (24%)	6 (20%)	0 (0%)	

## Discussion

Inflammation is a critical factor in the development and progression of CRC. The intricate relationship between chronic inflammation and cancer has been well-documented, with numerous studies highlighting how inflammatory processes contribute to tumorigenesis, tumor growth, and metastasis.

Chronic inflammatory conditions, such as inflammatory bowel disease, which includes Crohn’s disease and ulcerative colitis, significantly increase the risk of developing CRC. Persistent inflammation leads to a continuous cycle of damage and repair in the colonic epithelium, increasing the likelihood of genetic mutations and oncogenic transformations [15]. Inflammatory mediators, such as the vascular endothelial growth factor, enhance angiogenesis, providing tumors with the necessary blood supply for growth and metastasis. Increased vascular permeability, driven by cytokines such as IL-6 and tumor necrosis factor-α, facilitates the infiltration of cancer cells into lymphatic and blood vessels [16].

The GPS, which combines elevated serum CRP and decreased albumin concentration, is an inflammation-based marker that reflects the host’s systemic inflammatory response and metabolic stress. It has proven to be a valuable prognostic factor for survival in CRC patients [17]. Our study suggests the role of mGPS as a prognostic factor in patients with mCRC who did not undergo surgery. The prognostic value of mGPS remained statistically significant irrespective of treatment, molecular profile, or tumor burden. This has been confirmed by another retrospective study which included patients with CRC stage I-IV in which five-year OS rates were 35.2% vs. 74.9% vs. 92.6% [18].

An important prognostic factor for survival among patients diagnosed with CRC is the clinical status. The mGPS is an efficient and simple way to evaluate metabolic stress and, more precisely, malnutrition. A study by Richards et al. [19] reported that there is a significant connection between sarcopenia and a high mGPS. Another prospective study [20] reported that all patients with an mGPS of 2 were considered malnourished while only 19.1% of patients with an mGPS of 0 were considered malnourished according to the Subjective Global Assessment (SGA) scale. The correlation between mGPS and the three SGA categories (A, well nourished; B, moderately malnourished; C, severely malnourished) and GPS/mGPS score suggests inflammation’s role in cancer-induced cachexia. In our study, the results were similar with a majority of patients in the mGPS 2 cohort being underweight (BMI <18.5 kg/m^2^).

The role of mGPS as a predictive factor has been explored in several studies. Dréanic et al. [21] examined the predictive value of the mGPS in 49 patients with mCRC treated with 5-fluorouracil, cetuximab, and either oxaliplatin (60%) or irinotecan (30%). Patients with a GPS of 0 had significantly longer median PFS and OS compared to patients with mGPS of 1 and 2.

Similarly, Sharma et al. [22] studied 55 patients with mCRC undergoing first-line treatment with oral capecitabine monotherapy. Their findings revealed that mGPS, high CEA, and hypoalbuminemia were significant predictors of cancer-specific survival in univariate analysis. Patients with a GPS score of 2 had lower OS compared to those with GPS scores of 0 or 1.

This retrospective study investigating the role of the mGPS as a prognostic factor in mCRC has several inherent limitations that warrant consideration. First, retrospective studies are susceptible to selection bias due to their reliance on data from medical records, potentially leading to a non-representative sample of the broader patient population. This bias could influence the generalizability of findings beyond the study cohort. Second, the study is vulnerable to information bias stemming from the retrospective nature of data collection. Inaccuracies or incompleteness in medical records could affect the accuracy of mGPS scoring and other variables, potentially impacting the study outcomes. Moreover, the presence of confounding variables poses a significant challenge. Factors such as treatment history, performance status, comorbidities, and genetic mutations may not be uniformly captured or adjusted for in the analysis, potentially confounding the observed associations between mGPS and prognosis. Temporal ambiguity is another concern, as the timing and consistency of mGPS assessment relative to disease progression or treatment initiation may vary among patients, potentially influencing the prognostic value observed in the study.

Additionally, retrospective studies face limitations in establishing causality. While associations between mGPS and outcomes may be identified, causal relationships cannot be definitively inferred due to the study design's inherent constraints and the potential for unmeasured confounders. We acknowledge another limitation, notably the use of BMI, which is not the most accurate measure of body composition as it does not distinguish between muscle and fat mass or account for fat distribution. The cited studies used more precise methods such as anthropometric measurements and CT scans. Additionally, the small sample size of underweight patients in our study limits the generalizability and statistical power of our findings for this group. These factors highlight the need for future research with larger sample sizes and more accurate body composition assessments to validate our findings and provide a comprehensive understanding of the impact on patient outcomes.

## Conclusions

This study provides evidence that the mGPS may have prognostic value in mCRC irrespective of the treatment used. Our findings also suggest that mGPS is associated with BMI scores and potentially correlated with cancer-associated cachexia.
